# Expanding the Phenotypic Spectrum of Raynaud–Claes Syndrome: A Rett-like Presentation with Two New Cases

**DOI:** 10.3390/genes17060623

**Published:** 2026-05-29

**Authors:** Roberta Milone, Alessandro Orsini, Gemma Marinella, Valentina Rea, Rosa Pasquariello, Lorenza Marini, Roberta Battini

**Affiliations:** 1 Department of Developmental Neuroscience, IRCCS Fondazione Stella Maris, 56128 Pisa, Italy; 2 Pediatric Neurology, Pediatric University Department, Azienda Ospedaliera Universitaria Pisana, 56126 Pisa, Italy; aorsini.md@gmail.com (A.O.); lorenza.marini@ao-pisa.toscana.it (L.M.); 3 Department of Clinical and Experimental Medicine, University of Pisa, 56126 Pisa, Italy

**Keywords:** CLCN4-related neurodevelopmental disorder, X-linked intellectual disability, epilepsy, ASD

## Abstract

Pathogenic variants in the *CLCN4* gene are associated with a rare X-linked neurodevelopmental disorder, Raynaud–Claes syndrome, characterized by intellectual disability, epilepsy, language impairment, motor deficits, stereotypies, and structural brain abnormalities. Although heterozygous females are often considered to be only mildly affected, severe phenotypes have also been reported, and the clinical presentation shows considerable heterogeneity. The present study aims to summarize the current knowledge on CLCN4-related neurodevelopmental disorders through a review of the available literature and to describe two additional patients carrying pathogenic CLCN4 variants, one male and one female. The female patient was found to carry a de novo heterozygous variant (c.2152C > T), while the male patient harbored a de novo hemizygous variant (c.949G > A). Clinical data were compared with those reported in the literature in order to identify phenotypic similarities and differences among patients previously described with the same mutations. Furthermore, in light of the literature review and the clinical data collected from our patients, we propose considering Raynaud–Claes syndrome as a Rett-like condition. This perspective expands the scope of differential diagnosis and underscores the importance of multidisciplinary and longitudinal diagnostic evaluation to improve clinical characterization, therapeutic management, and genetic counseling.

## 1. Introduction

Raynaud–Claes syndrome (MRXSRC, MIM#300114), also known as CLCN4-related neurodevelopmental disorder, is a rare X-linked condition caused by pathogenic variants in the *CLCN4* gene located on chromosome Xp22.2 [1].

This disorder has been associated with both loss-of-function (LoF) and gain-of-function (GoF) variants in *CLCN4* [2]. Missense variants appear to be absolutely prevalent among those reported, although frameshift, truncating, splice-site, and nonsense variants have also been described [1,2,3,4,5,6,7,8,9,10,11]. Missense variants are related to a more severe phenotype, both regarding functional aspects and epilepsy outcome, while frameshift or intragenic deletion variants were associated with relatively mild pictures compared with the majority of individuals with missense variants [10].

Pathogenic variants in *CLCN4* are associated with global developmental delay, intellectual disability (ID) ranging from mild to profound, and a spectrum of seizure types that may be difficult to control. Additional common features include autistic behaviors, anxiety or aggression, movement abnormalities (such as ataxia or dystonia), microcephaly, gastrointestinal dysmotility, and variable dysmorphic features [11].

*CLCN4* encodes the voltage-dependent Cl^−^/H^+^ exchanger ClC-4, which is essential for neuronal morphogenesis and dendritic maturation, as well as for endosomal ion homeostasis, intracellular vesicle trafficking, degradation, and autophagy—all processes that are critical for neuronal function and survival [2,11].

The first description of this syndromic X-linked condition dates back to 1996, when Raynaud et al. reported a two-generation French family with five affected males presenting severe to profound intellectual disability and variable behavioral difficulties [12]. In the same year, Claes et al. described a Belgian family with five affected males across two generations, characterized by intellectual disability, challenging behaviors, and autistic features [13]. Heterozygous females in these families were either neurotypical or exhibited mild neurocognitive or psychiatric manifestations. Based on these observations, a distinct entity of X-linked intellectual disability, later termed Raynaud–Claes syndrome, was proposed.

The association between *CLCN4* mutations and neurodevelopmental disorders was first reported in 2013, when a de novo loss-of-function mutation was linked to epileptic encephalopathy and severe developmental delay. Three years later, protein-truncating and missense variants identified in five unrelated families were also associated with X-linked intellectual disability [4].

To date, approximately 130 individuals from 75 families with *CLCN4*-related disorders have been described in the literature, encompassing more than 70 distinct variants of this gene [2].

Despite the growing number of identified cases, genotype–phenotype correlations remain only partially understood, and *CLCN4*-related disorders are still frequently underdiagnosed. Notably, heterozygous females may present with severe neurodevelopmental impairment, including profound deficits in verbal communication and social interaction [1,2,3,4,5,6,7]. This observation challenges the traditional assumption that females are typically only mildly affected in X-linked disorders.

In this narrative review, we collected reported *CLCN4* mutations and their associated phenotypes in order to summarize the current knowledge on *CLCN4*-related neurodevelopmental disorders. Additionally, we describe two new patients carrying pathogenic *CLCN4* variants, with the aim of comparing them to previously reported cases, refining genotype–phenotype correlations, and expanding the known phenotypic spectrum. We also provide detailed longitudinal clinical, neurophysiological, neuroimaging, ophthalmological, and rehabilitative data.

Finally, we propose that *CLCN4* missense mutations may underlie a Rett-like phenotype.

## 2. Methods

We conducted a systematic literature review on *CLCN4*-related neurodevelopmental disorders by searching the PubMed database using the keywords “*CLCN4* mutation,” “Raynaud–Claes syndrome,” and “*CLCN4*-related neurodevelopmental disorder.”

In addition, we evaluated two patients at our Department of Developmental Neuroscience who presented with developmental delay, significant language impairment, intellectual disability, and epilepsy. Genetic analyses were performed after obtaining informed consent from the patients’ legal guardians and included array comparative genomic hybridization (array-CGH), target multigene panel for epilepsy and brain malformations, and clinical exome sequencing in both cases.

The genomic DNA of the patients was isolated from peripheral blood by standard methods. Array-CGH analysis was performed in both patients using the Agilent 8 × 60 K microarray oligonucleotide platform with a median resolution of 100 Kbp, following manufacture’s protocol (Agilent Technologies, Santa Clara, CA, USA). DNA from healthy subjects (one male and one female) was used as the control (Agilent Technologies, Santa Clara, CA, USA). The genomic imbalance coordinates refer to the Genome Reference Consortium Human Build 37 (GRCh37/hg19).

Target multigene panel including 373 genes associated with epilepsy was performed in both patients. Target enrichment and library preparation were performed using a custom-designed Paired-End 150 bp on NextSeq (Illumina, San Diego, CA, USA). Variants were annotated and filtered using the ANNOVAR tool (version 20200316). Putative causative variants were analyzed by Sanger sequencing to confirm the next-generation sequencing (NGS) results in probands and investigated in the parents to check the inheritance status. We classified variants according to the international guidelines of the American College of Medical Genetics and Genomics (ACMG) Laboratory Practice Committee Working Group [14].

Whole exome sequencing (WES) has been performed in patient 1 using paired-end technology (150 bp) on the Illumina NextSeq 500 platform, following enrichment of the coding regions of the human genome using a clinical exome kit (SureSelect XT2 Clinical Research Exome, Agilent Technologies, Santa Clara, CA, USA and/or Twist Human Core Exome Kit, Twist Bioscience, South San Francisco, CA, USA), in accordance with the manufacturer’s specifications. The quality of the libraries and sequencing was assessed using standard metrics, with an average coverage of target regions exceeding 100×. The following quality parameters were achieved: >95% of target bases covered at ≥20× and >91% at ≥30×.The sequences were aligned to the human reference genome (GRCh37/hg19) using BWA-mem (v0.7.17). Variant calling was performed using GATK HaplotypeCaller (v4.0.2.1). Variants annotation and filtering were carried out using VarSeq (Golden Helix, Bozeman, MT, USA) and proprietary software (Personal Genomics, University of Verona), followed by the removal of sequencing and alignment artifacts. Non-synonymous exonic variants and variants at canonical splicing sites (±2 nucleotides from the exons) with an allelic frequency of less than 1% in major population databases were included in the analysis. Variants were interpreted in accordance with the guidelines of the American College of Medical Genetics and Genomics (ACMG) and the Association for Molecular Pathology (AMP) [14]. Pathogenic, likely pathogenic or variants of uncertain significance (VUS) were reported and, where indicated, confirmed by Sanger sequencing. Variant filtering was guided by the proband’s clinical phenotype. The analysis was performed in a trio-based context (proband and parents).

WES has been performed in patient 2 using the SureSelect Human All Exon V7 kit (Agilent Technologies, Santa Clara, CA, USA), which covers approximately 60 Mb of the coding regions of the human genome. Paired-end sequencing (150 bp) was performed on a NovaSeq 6000 platform (Illumina Inc., San Diego, CA, USA), in accordance with the manufacturer’s instructions. Sequencing quality was assessed using standard metrics, with ≥20× coverage of at least 98% of the target regions. Raw reads were aligned to the human reference genome (GRCh37/hg19) using the Burrows–Wheeler Aligner (BWA, v0.7.15). Variants calling was performed using the Haplotype Caller algorithm from the Genome Analysis Toolkit (GATK, v4.0). Variant annotation was carried out using Variant Interpreter (v2.6). Variants were filtered to exclude sequencing artifacts and prioritized according to the following criteria: (i) location in exonic regions or canonical splicing sites (±15 nucleotides from exon–intron boundaries); (ii) predicted functional impact (non-synonymous variants); (iii) minor allele frequency < 1% in population databases, including the Genome Aggregation Database (gnomAD); and (iv) read depth ≥ 20×. A trio-based analysis was performed to assess inheritance patterns, including de novo, autosomal recessive and X-linked variants. Variants that were inconsistent with the expected segregation pattern were excluded. Variants prioritization focused on genes known to be associated with the proband’s phenotype. The classification and interpretation of variants were carried out in accordance with the guidelines of the ACMG and the AMP [14].

Clinical assessment included a detailed neurodevelopmental history, comprehensive neurological examination, and structured cognitive and adaptive evaluations using the Griffiths Developmental Scales (third edition), the Bayley Scales of Infant and Toddler Development (third edition), and the Vineland Adaptive Behavior Scales, Second Edition [15,16,17]. Additional evaluations comprised behavioral and ophthalmological assessments, as well as longitudinal neuroimaging.

Epilepsy was characterized through detailed clinical descriptions of seizure types, serial electroencephalogram (EEG) and video-EEG recordings, and documentation of pharmacological management. All assessments were conducted longitudinally to capture developmental trajectories. Rehabilitation programs were also documented, including physical, occupational, speech, and educational intervention.

## 3. Results

Literature Review: See Table 1 and Table 2A,B.

## 4. Case Presentations

### 4.1. Patient 1

Patient 1 is a 9-year-6-month-old female born at term, small for gestational age. At birth, weight was 2520 g, length 45 cm, and occipito-frontal circumference (OFC) 32.5 cm. Apgar scores were 9 at 1 min and 10 at 5 min. Intrauterine growth restriction was detected at 32 weeks of gestation.

Motor development was markedly delayed: head control was achieved at 6 months, independent sitting at 13 months, crawling at 14 months, and autonomous ambulation at 2 years. Language development was severely impaired, with absence of early verbal output; babbling and vowel vocalizations emerged at 3 years without further progression. Request pointing appeared at 9 years, and no additional communicative gestures were observed.

**Figure 1:**
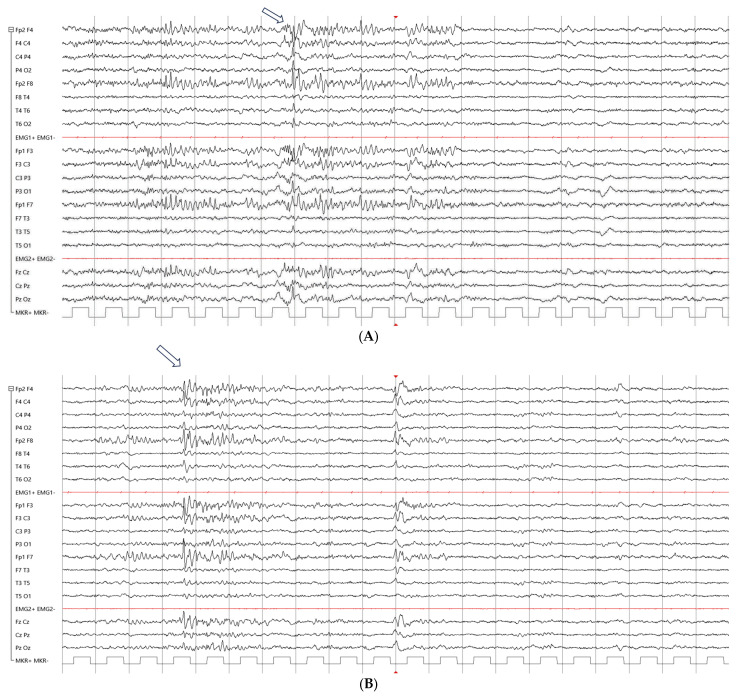
Polygraphic-EEG recording (10–20 International System, 20 s/page, low-pass band filter: 70 Hz, high-pass-band filter: 1 Hz, gain: 150 mcV/cm). Anterior bilateral spikes (white arrow) during drowsiness (**A**) and sleep (**B**).

Cognitive assessment at 23 months, performed using the Bayley Scales of Infant and Toddler Development, Third Edition, showed a developmental level below 12 months, with absence of object permanence. At 9 years and 6 months, formal cognitive assessment was not feasible. Adaptive functioning, assessed with the Vineland Adaptive Behavior Scales, Second Edition (VABS-II), was consistent with profound intellectual disability (Adaptive Quotient [AQ] 20), while socialization (AQ 31) and daily living skills (AQ 26) were in the severe intellectual disability range. Epilepsy onset occurred at 4 months, with clusters of episodes characterized by hypertonia and staring during febrile episodes; initial EEG was normal. At 15 months, a generalized seizure with gaze deviation was observed, followed by recurrent seizures during febrile episodes. From 18 months, additional events occurred, characterized by erratic clonic movements and head deviation followed by hypotonia, in both febrile and afebrile conditions. EEG recordings showed normal background activity during wakefulness and diffuse fast activity with anterior predominance and mild paroxysmal abnormalities during sleep. Treatment with valproate (up to 500 mg/day) resulted in seizure remission from 2 years of age. At 9 years and 6 months, EEG demonstrated anterior theta activity and diffuse epileptiform abnormalities during sleep (Figure 1).

Neurological examination revealed independent ambulation with reduced motor fluidity and gait instability, including a tendency to equinus foot posture. Muscle tone and strength were normal. Deep tendon reflexes were brisk in the lower limbs, with bilateral extensor plantar responses and no clonus. Microcephaly was present (OFC 46 cm, <1st percentile).

Behavioral features included frequent stereotypies (midline movements, hand-to-mouth behaviors) and bruxism, with markedly impaired functional use of objects. Verbal comprehension was limited to simple, context-supported commands, and response to name was inconsistent.

Ophthalmological evaluation revealed exotropia, hypermetropia, reduced visual acuity, and absence of stereopsis. Early feeding difficulties included impaired swallowing of liquids and gastroesophageal reflux.

Brain MRI at 3 years showed bilateral fronto-parietal and periventricular white matter hyperintensities and thinning of the corpus callosum.

Previous genetic investigations included array-CGH, which was negative, and a targeted epilepsy gene panel identifying a heterozygous *FLNA* variant of uncertain significance (c.7144G > C, p.Val2382Leu), inherited from her father. WES subsequently identified a de novo heterozygous pathogenic variant in *CLCN4* (c.2152C > T, p.Arg718Trp).

The variant c.2152C > T has been classified as pathogenic based on aggregated data from public databases, according to ACMG guidelines (https://franklin.genoox.com/clinical-db/variant/snp/chrX-10188877-C-T, accessed on 5 May 2026). Specifically, functional studies support the PS3 criterion (pathogenic strong: well-established functional studies demonstrate a damaging effect on the gene or gene product). As the variant occurred de novo, PS2 (pathogenic strong) was applied. Population data support PM2 (pathogenic moderate: extremely low frequency in gnomAD population databases). In silico predictions support PP3 (pathogenic supporting: computational tools unanimously predict a deleterious effect). Additionally, PM5 (pathogenic moderate) was assigned, as a different amino-acid change at the same residue has been previously reported as pathogenic.

The patient is currently enrolled in a comprehensive multidisciplinary rehabilitation program, including speech therapy, neuropsychomotor therapy, occupational therapy, and full-time educational support. Gradual improvements in attention and shared attention have been observed in structured settings despite severe communication impairment.

### 4.2. Patient 2

Patient 2 is a 6-year-old male born at 41 + 5 weeks of gestation. At birth, weight was 3900 g, length 52 cm, and OFC 36 cm. Due to a double nuchal cord, ventilatory support was required at birth. Apgar scores were 6 at 1 min and 8 at 5 min.

Motor development was delayed: independent sitting was achieved at 12 months, standing at 15 months, and autonomous ambulation at 20 months. Language development was also delayed, with babbling at 15 months and first words at 2 years. Receptive language corresponded to approximately 17 months of age, and at 6 years expressive language consisted mainly of two-word combinations.

Cognitive assessment at 30 months, performed using the Bayley Scales of Infant and Toddler Development, Third Edition, and the Griffiths Developmental Scales, Third Edition, showed a developmental level corresponding to a mental age of 18 months. Re-evaluation at 6 years with the Griffiths scales indicated a developmental level of approximately 24 months. Adaptive functioning (VABS-II) was consistent with moderate-to-severe intellectual disability (AQ 34), with more pronounced impairment in motor (AQ 28) and communication (AQ 38) domains, while socialization (AQ 57) and daily living skills (AQ 59) were in the mild intellectual disability range.

Epilepsy onset occurred at 11 months with an apparently generalized seizure during a febrile episode, followed by clusters of myoclonic jerks predominantly affecting the upper limbs, mainly during febrile illnesses. A further febrile myoclonic seizure was documented at 5 years. He had never assumed chronic pharmacotherapy, only clonazepam and diazepam at the occurrence. EEG and video-EEG recordings showed slowed background activity with angular slow waves over the occipital-temporal regions, predominantly on the right, persisting during sleep, with rare sharp waves in the right posterior temporal region and no clear epileptiform paroxysms.

Neurological examination showed generalized hypotonia, ligamentous laxity, valgus-pronated feet, and marked impairment in both gross and fine motor skills. OFC was within normal limits.

Behavioral features included attention deficit and hyperactivity.

Ophthalmological evaluation revealed an immature visual profile, with central hyperfixation, non-smooth visual pursuit, dysmetric saccades, occasional mild vertical misalignment, reduced contrast sensitivity (down to 2.5%), and preserved visual acuity for age. Feeding difficulties with liquids were reported during the first year of life.

**Figure 2:**
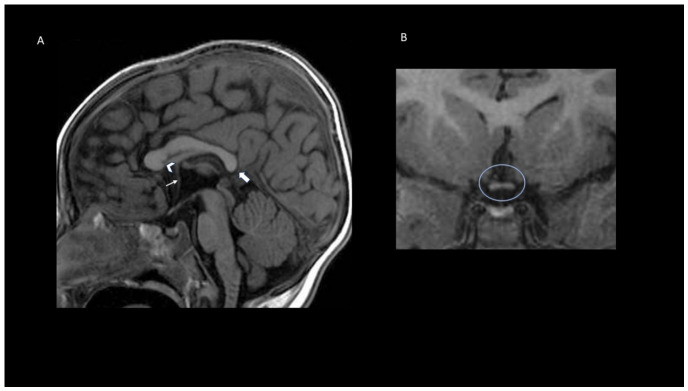
(**A**) Sagittal T1-weighted image demonstrates a reduced anteroposterior diameter of the corpus callosum, hypoplasia of the splenium (thick arrow), poor visualization of the rostrum (arrow-head), and marked thinning of the anterior commissure (thin arrow). (**B**) The circle in coronal T1-weighted image demonstrates mild thinning of the optic chiasm.

Brain MRI at 1 year demonstrated a mildly shortened corpus callosum with an unrecognizable rostrum and mild ectasia of the temporal horns associated with incomplete hippocampal inversion. Follow-up MRI at 5 years confirmed dysmorphic temporal horns with hippocampal eversion, corpus callosum abnormalities, a thin optic chiasm (Figure 2), and small focal subcortical and juxtacortical white matter hyperintensities.

Neurometabolic investigations were unremarkable.

Array-CGH was normal. Genetic testing included a targeted epilepsy gene panel identifying variants of uncertain significance in *CACNA1A* (maternally inherited), *ATP1A2*, and *GRIN1* (paternally inherited), with negative *SCN1A* analysis. WES identified a de novo hemizygous likely pathogenic variant in *CLCN4* (c.949G > A, p.Val317Ile), confirmed by parental testing.

The variant c.949G > A has been classified as likely pathogenic based on aggregated data from public databases, according to ACMG guidelines (https://franklin.genoox.com/clinical-db/variant/snp/chrX-10176190-G-A, accessed on 5 May 2026). Specifically, de novo occurrence supports PS2 (pathogenic strong). Population data support PM2 (pathogenic moderate: extremely low frequency in gnomAD population databases). Functional considerations support PP2 (pathogenic supporting: missense variant in a gene with a low rate of benign missense variation, where missense variants are a common disease mechanism). Additionally, this variant has been recently reported as pathogenic by reputable source data, supporting PP5.

The patient is currently enrolled in a multidisciplinary rehabilitation program, including psychomotor therapy, speech and language therapy, physiotherapy, and full-time educational support. Plantar orthoses are in use, and ongoing interventions target motor, cognitive, communicative, and social domains.

## 5. Discussion

Pathogenic variants in the *CLCN4* gene are increasingly recognized as a cause of rare X-linked neurodevelopmental disorders, affecting both hemizygous males and heterozygous females [2,5,6]. Across reported cases, a relatively consistent neurodevelopmental phenotype emerges, characterized by global developmental delay, ID of variable severity, heterogeneous epileptic manifestations, motor impairment, and behavioral disturbances, often meeting diagnostic criteria for attention deficit hyperactivity disorder (ADHD) and autism spectrum disorder (ASD).

In males, the most common clinical feature is moderate-to-severe ID. However, a male with a normal verbal Intelligence Quotient (IQ) has been reported in association with the p.(Phe319Ser) missense loss-of-function (LoF) mutation (Table 1, [6]). Predicting cognitive outcomes in females with *CLCN4*-related conditions remains challenging, as the phenotype spans a wide spectrum, ranging from severely affected individuals to apparently asymptomatic carriers [6]. Notably, and in contrast to what is typically observed in X-linked disorders, females carrying de novo variants may exhibit a severity comparable to that of affected males [16]. Furthermore, cognitive, linguistic, and autistic regression has been described in both sexes and appears to be independent of the specific genetic variant, mutation type, or the presence of epilepsy (Table 1).

Epilepsy represents a major feature of *CLCN4*-related neurodevelopmental disorders and appears to be up to three times more frequent in males than in females (Table 1, [6,8]). The most common seizure types include focal seizures and generalized tonic–clonic seizures, followed by infantile spasms, myoclonic seizures, atypical absences, eyelid myoclonus, tonic and atonic seizures, myoclonic–atonic seizures, and Lennox–Gastaut syndrome (Table 1, [6,8]). Seizures may also be triggered by fever.

Most patients with *CLCN4*-associated epilepsy present with moderate-to-profound ID and marked language impairment. Pharmacoresistant epilepsy may negatively affect cognitive outcomes [2]. However, in most cases, seizure control does not lead to significant cognitive improvement, suggesting that *CLCN4* dysfunction may directly cause irreversible neurodevelopmental impairment independently of epileptic activity [6,8,10]. This hypothesis is supported by evidence that *CLCN4* mutations disrupt dendritic development during neuritogenesis, potentially impairing synaptic function and altering neurodevelopment irrespective of seizures [10].

Consistent with these observations, both of our patients—one female and one male—carrying de novo variants (heterozygous c.2152C > T, p.(Arg718Trp), and hemizygous c.949G > A, p.(Val317Ile), respectively) presented with early-onset epilepsy within the first year of life. Seizures were frequently associated with fever or intercurrent inflammatory events and showed variable semiology. In both patients, seizures were well controlled during early childhood. Despite this, their overall clinical presentations differed significantly: the female patient was more severely affected than the male and showed a phenotype more consistent with that reported in individuals carrying the same genetic variant, regardless of epilepsy status (Table 2A,B). In both cases, developmental delay preceded seizure onset, in line with previous reports [10]. Moreover, moderate-to-severe ID has been described even in patients without epilepsy, while individuals with borderline cognitive impairment or normal verbal IQ may present with seizures (Table 1, [6]).

Both variants identified in our patients have been previously reported, and their phenotypes are consistent with those described in the literature.

The p.(Arg718Trp) variant identified in our female patient has been reported in nine additional individuals (four females, two males, and three of unknown sex) [6,10,11,18] (Table 2A). This recurrent missense variant likely represents a mutational hotspot in *CLCN4*, as previously suggested [10]. It has been classified as a missense LoF variant [6] and, similarly to other variants of this type, can result in severe clinical manifestations in both sexes [9]. Among reported female patients (Table 2B), one shared features with our patient, including epilepsy, ID, ASD, and significant language impairment [6]. Other reported cases include a female with severe ID, regression, and absence seizures [11], and another with hypotonia, epilepsy, ASD, feeding difficulties, and verbal dyspraxia (ID not specified) [19]. Our patient additionally presented with microcephaly. Two male patients with this variant also showed early-onset epilepsy (Table 2A, [6,11,18]). De novo occurrence was confirmed in three previously reported females as well as in our patient, while inheritance was unknown in one case (Table 2B).

The male patient in our cohort carried the p.(Val317Ile) variant, previously reported in four other male individuals ([6,19], Table 2A,B). All exhibited moderate-to-severe ID; epilepsy and ADHD were present in two out of four cases, autistic features in two, and swallowing difficulties in three. Hypotonia, delayed ambulation, and language delay were observed in all patients, with one remaining non-verbal. None presented with microcephaly. Neuroimaging consistently revealed corpus callosum abnormalities (dysplastic or hypoplastic), and three out of four patients also showed a thin optic chiasm or optic atrophy. These findings, also observed in our patient, suggest a possible preferential involvement of visual pathways associated with this variant (Table 2B, [6]), which may thus have a remarkable and recognizable neuroimaging pattern. De novo occurrence was confirmed in three previously reported males as well as in our patient, while another male inherited the variant from his mother (Table 2B, [6,19]).

As previously noted, de novo variants tend to be associated with more severe phenotypes compared to inherited variants [10].

The p.(Val317Ile) variant has been classified as a missense gain-of-function (GoF) variant [6]. Although the most functionally severe GoF *CLCN4* variants have been reported exclusively in females—likely due to embryonic lethality in males [6,9]—the functional impact of this specific variant appears to be milder [6]. Feeding difficulties, commonly observed in patients with GoF variants [2,6], were also present in our patients (especially swallowing difficulties), with the female patient carrying instead a LoF variant. These difficulties may reflect broader oral–motor dysfunction, consistent with the prominent language impairment observed in *CLCN4*-related disorders, which ranges from complete absence of speech to phonetic and articulatory deficits [19].

In a study specifically investigating speech and language abilities, a female patient carrying the same p.(Arg718Trp) variant presented with verbal apraxia, dysarthria, slow speech rate, limited vocabulary, and comprehension difficulties, along with feeding issues, epilepsy, ASD, hypotonia, and mega cisterna magna [19]. In contrast, our patient was non-verbal, suggesting an even more severe language impairment despite sharing the same mutation. In the same study, a male patient sharing the same p.(Val317Ile) variant of ours, exhibited slow and unclear speech, limited vocabulary, social difficulties, feeding issues, hypotonia, agenesis of the corpus callosum, and cerebellar vermis hypoplasia [19].

Among other key features of *CLCN4*-related disorders, ADHD and ASD (or autistic traits) are common comorbidities, affecting approximately 60% and 55% of males, respectively, and about 47% and 40% of females with de novo variants [1,2,6,11,20]. In our cohort, the female patient exhibited severe communicative and relational impairment with stereotypies, whereas the male patient presented with ADHD. Progressive microcephaly, more frequently reported in females with de novo variants (approximately 80% vs. 20% in males) [6], was observed in our female patient but not in the male. Progressive spasticity, reported in about 40% of affected females, was also present in our female patient, who showed pyramidal signs. Developmental regression has been described in both sexes, independent of variant type or functional mechanism (LoF vs. GoF).

Based on the available literature and the detailed clinical characterization of our patients, we propose that CLCN4-related neurodevelopmental disorders may fall within the spectrum of Rett-like conditions. This umbrella term encompasses not only the classical Rett phenotype but also atypical presentations, such as congenital Rett variants (e.g., *FOXG1* mutations) and overlapping Rett-like phenotypes (e.g., Pitt–Hopkins syndrome, Christianson syndrome, Angelman syndrome, *STXBP1* mutations, *PURA* mutations, *MEF2C* mutations), in which regression is not always present [23] (Table 3).

Key overlapping features include developmental regression (although not invariably present), microcephaly, intellectual disability, severe language impairment, ASD with midline stereotypies (including hand stereotypies and hand-to-mouth behaviors), epilepsy, a history of hypotonia, and pyramidal signs with ataxic gait [24].

**Figure 3:**
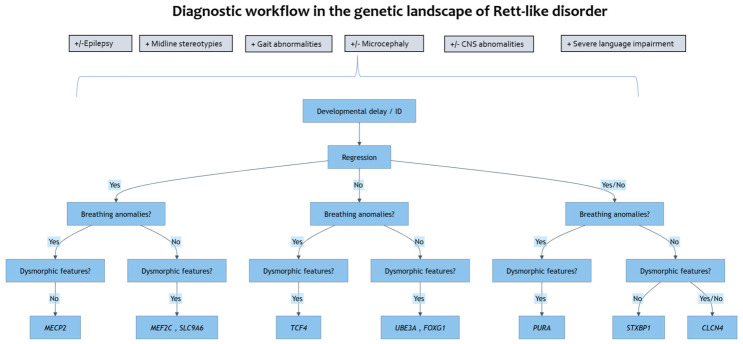
Legend and abbreviation: CNS: Central Nervous System; ID: Intellectual Disability.

This perspective broadens the differential diagnosis and highlights the importance of multidisciplinary and longitudinal evaluation to improve clinical characterization, management, and genetic counseling. In order to facilitate the differential diagnosis among Rett-like conditions, including *CLCN4*-related disorders within this spectrum, we have proposed a graphical diagnostic workflow (Figure 3).

Further detailed clinical reports are needed to better define the phenotypic spectrum of this rare disorder.

## 6. Conclusions

*CLCN4*-related neurodevelopmental disorder is a rare X-linked condition with highly variable clinical manifestations, including intellectual disability, epilepsy, motor and language impairment, behavioral and psychiatric features, and ophthalmological abnormalities. The two patients described here expand the known phenotypic spectrum, particularly by highlighting a severe early-onset neurodevelopmental and communicative phenotype in a heterozygous female.

Although epilepsy is a frequent feature, it shows variable onset and semiology, with persistent EEG abnormalities reflecting underlying network instability. Structural neuroimaging often reveals subtle abnormalities that do not reliably predict functional outcomes, supporting the hypothesis that *CLCN4* variants primarily affect neuronal connectivity and synaptic organization.

Our findings underscore the importance of early recognition, comprehensive longitudinal assessment, and individualized multidisciplinary management, including neurodevelopmental, neurophysiological, neuroimaging, behavioral, ophthalmological, and rehabilitative evaluations and also support the hypothesis that *CLCN4*-related disorders should be considered within the spectrum of Rett-like neurodevelopmental conditions, independently of epilepsy severity. By integrating detailed clinical observations with existing literature, this study contributes to refining genotype–phenotype correlations and supports the inclusion of *CLCN4*-related disorders within the spectrum of Rett-like conditions.

## Figures and Tables

**Table 1:** Summary of *CLCN4* genetic variants and their corresponding phenotypic descriptions reported in the literature and in this study.

Genetic Variant	Type of Variant	Gender	Age (Years if not Otherwise Specified)	Epilepsy	Neurologic Signs	Intellectual Disability	Language	ASD/Autistic Traits	Dysmorphisms	Brain MRI	Reference
c.1390-12(IVS9) T > G	Splicing	M	6	No	No	Yes (not specified the level)	Non-verbal	Yes	No	Right temporal arachnoid cyst and slightly wide left temporal subcranial plate gap	[1]
c.1576 + 5G > A	Splicing	M	19	Yes	walking	Borderline	Sentences (regression)	Yes	NR	NA	[2]
p.(Ala448Val)	Missense LoF	F	3	No	Hypotonia, walking at 28 months	Severe	Non-verbal	Yes	Bushy eyebrows, downslanted palpebral fissures, esotropia, depressed nasal bridge, sparse teeth	Thin corpus callosum, mega cisterna magna, ventriculomegaly	[5]
p.(Ala555Val)	Missense GoF	F	3	No	Hypertonia, walking at the age of 2	Moderate	Severe delay	No	Microcephaly	Widened bilateral frontal-temporal extra brain space and enlarged the left ventricle	[1]
p.(Ala555Val)	Missense GoF	F	5	No	Hypertonia, non-ambulatory, swallowing difficulties	Severe	Non-verbal	No	Microcephaly	Small cranial volume, slightly thin the posterior of the corpus callosum, and widened ventricles	[1]
p.(Ala555Val)	Missense GoF	F	9	No	Non-ambulatory, swallowing difficulties with gastrostomy feeding	Yes, not specified the level	Non-verbal	No	Microcephaly	Diffuse cortical volume loss with mild lateral and third ventricular enlargement	[6]
p.(Ala555Val)	Missense GoF	F	4	No	Non-ambulatory, swallowing difficulties with gastrostomy feeding	Moderate	Non-verbal	Stereotypical hand movements	Microcephaly, positional plagiocephaly, deep-set and wide-spaced eyes, broad bulbous nose, large ears, small jaw, and high palate	Agenesis of the corpus callosum and anterior commissure (complete commissural agenesis) and abnormal orientation of the hippocampi	[6]
p.(Ala555Val)	Missense GoF	F	4	No	Non-ambulatory feeding difficulties	Yes, not specified the level	Non-verbal	No	Microcephaly	Small pons, immature myelination	[6]
p.(Ala555Val)	Missense GoF	F	10	No	Hypotonia, walking at 18 months	Yes, not specified the level	Two-to-three-word phrases	No	Microcephaly, posteriorly rotated ears, slightly arched eyebrows, slightly depressed nasal bridge, decreased muscle bulk, prominent columella, fifth finger clinodactyly	Normal	[6]
p.(Ala555Val)	Missense GoF	F	18 months	No	Hypertonia, Non-ambulatory	Severe	Non-verbal	NR	Microcephaly, depressed nasal bridge	Dilation of the cerebral ventricles, thin corpus callosum, delayed myelination	[2]
p.(Ala555Val)	Missense GoF	F	5	No	Walking	Severe	Few words	NR	NR	NA	[2]
p.(Ala555Val)	Missense GoF	F	28	No	Walking	Severe	Simple sentences	NR	NR	NA	[2]
p.(Arg41Trp)	Missense LoF	M	4	No	Non-ambulatory, autonomic, paroxysmal involuntary eye movements, tic disorder	Severe	Single words	NR	NR	NA	[2]
p.(Arg360Ser)	Missense LoF	M	18	No	No	Severe (regression)	Non-verbal (regression)	Yes	Elongated face, long nose, mildly anteverted ears, prominent chin, and a pre-auricular pit at base of left helix. 5th finger proximal interphalangeal joint camptodactyly, mild pectus excavatum, thoracolumbar kyphosis and mild scoliosis with leg length discrepancy	Non-specific bilateral small punctate frontal white matter hyperintensities, mildly prominent Virchow-Robin spaces. Slightly bulky corpus callosum	[6]
p.(Arg603Trp)	Missense LoF	M	2	Infantile spasms, focal seizures	Non-ambulatory	Severe	Non-verbal	No	Depressed nasal bridge, small chin	Enlargement of subarachnoid spaces, dilation of the cerebral ventricles	[2]
p.(Arg694Gln)	Missense LoF	M	5	No	Feeding difficulties, poor vision	Yes (not specified the level)	Non-verbal	Traits	No	Normal	[1]
p.(Arg718Trp)	Missense LoF	F	6	Yes, blank stares, cyanosis, and emesis	No	Yes (not specified the level, regression)	Very limited expressive language	Yes, echolalia, stereotyped or repetitive motor movements,	No	NA	[6]
p.(Arg718Trp)	Missense LoF	F	9	Hypertonia and staring during hyperpyrexia, erratic clonic movements and leftward head deviation followed by diffuse hypotonia, occurring during febrile and afebrile states	Hypotonia, walking at 24 months, gait instability, pyramidal signs, feeding difficulties	Profound (regression)	Non-verbal	Yes, midline stereotypies, hand-to-mouth movements, bruxism	Microcephaly	Bilateral frontal-parietal and periventricular white matter hyperintensities and corpus callosum thinning	This study
p.(Arg718Trp)	Missense LoF	F	8	Absence seizures	Hypotonia	Severe, regression	NR	NR	NR	NR	[11]
p.(Arg718Trp)	Missense LoF	F	NR	NR	NR	NR	NR	NR	NR	NR	[6]
p.(Arg718Trp)	Missense LoF	M	1 month	Intractable epilepsy	NR	Severe	NR	NR	NR	NR	[10]
p.(Arg718Trp)	Missense LoF	M	3	Intractable epilepsy	NR	NR	NR	NR	NR	NR	[18]
p.(Arg718Trp)	Missense LoF	NR (3)	NR	NR	NR	NR	NR	NR	NR	NR	[6]
p.(Arg718Trp)	Missense LoF	F	4	Yes	Hypotonia, feeding difficulties	NR	First words at 12–15 months, CAS, dysarthria	Yes	NR	Mega cisterna magna	[19]
p.(Asn141Ser)	Missense LoF	M	3	No	NR	Moderate	Non-verbal	Yes	NR	NA	[1]
p.(Asn309Ser)	Missense LoF	M	13	Possible absence seizures	Hypotonia, walking at the age of 23 months, ataxia	Moderate-severe	Sentences	Yes	Long face with square chin and upslanting palpebral fissures, long fingers, pes planus, and joint hyperextensibility	Complete agenesis of the corpus callosum	[6]
p.(Asn309Ser)	Missense LoF	M	21	Focal seizures with secondary generalization	Hypotonia, ataxia	Moderate-severe	NR	No	Long face with square chin, distinct philtrum and tented upper lip, bilateral strabismus, long fingers, camptodactyly pes planus, and joint hyperextensibility	Complete agenesis of the corpus callosum	[6]
p.(Asp15Serfs*18)	Frameshift	M (5)	NR	NR	NA	Borderline (1/5), Mild (3/5), Moderate (1/5)	NR	No	No	NA	[4]
p.(Gln392Ter)	Nonsense	M	4	No	NR	Global developmental delay	Speech disorder	NR	Yes	NA	[7]
p.(Gln489Lys)	Missense LoF	F	32	No	Migraines, tremors, poor balance	No, IQ 131 (WAIS-IV)	Normal	Traits	No	Rathke cleft cyst	[6]
p.(Glu280Asp)	Missense LoF	M	8	Tonic–clonic and focal seizures, associated with fever or not	Hypotonia	Moderate	Sentences	Yes	No	NA	[6]
p.(Gly269Asp)	Missense LoF	F	15	No	Hypotonia, swallowing difficulties requiring gastrostomy	Mild	First words at 30 months	No	Microcephaly, elongated and narrow face, sloping forehead, prominent nose with high nasal bridge, long philtrum, thin upper lip vermillion, micrognathia, high arched palate, dental crowding, and bilateral 5th finger clinodactyly, spina bifida occulta	Mild prominence of the lateral ventricles with septation through the right lateral ventricle at the base of the frontal horn	[6]
p.(Gly342Arg)	Missense LoF	M	5	Focal epilepsy with recurrent status epilepticus	Walking with support	Moderate	8 words	Yes	No	Small foci of T2-FLAIR hyperintensity, mostly within the bifrontal white matter (prominent perivascular spaces)	[8]
p.(Gly342Arg)	Missense LoF	M	2	Tonic and versive seizures with loss of consciousness, easily triggered by fever, often presenting in clusters	No	Mild	Sentences	No	No	Slightly widening of the left ventricle	[20]
p.(Gly342Glu)	Missense LoF	M	3	One focal seizure	Walking at the age of 2 years	Yes, not specified the level	3 inconstant words	No	No	Normal	[6]
p.Gly480Arg	Missense LoF	M	2	Febrile seizures	Walking	Moderate	Non-verbal (regression)	No	No	Normal	[2]
p.(Gly484Arg)	Missense LoF	NR	NR	NR	NR	NR	NR	NR	NR	NR	[6]
p.(Gly526Ser)	Missense LoF	M	17	Focal onset seizures with secondary generalized seizures and myoclonic seizures	Severe feeding difficulties	Mild	NR	NR	Microcephaly, hypoplastic upper maxilla and dental malocclusion	NA	[6]
p.(Gly544Arg)	Missense LoF	F	24	Myoclonic, tonic–clonic seizure, atypical absences, and focal impaired awareness seizures	Hypotonia, walking at 18 months	Yes, not specified the level	Non-verbal	Autistic regression	Obesity, short stature, round face, round and flat nasal tip, fleshy lips, and posteriorly rotated ears, small hands and feet	Asymmetry of the mesial temporal lobes and lateral ventricles due to a smaller size of the left hippocampus	[21]
p.(Gly544Arg)	Missense LoF	M	NR	Early onset epileptic encephalopathy	NR	Severe	NR	NR	NR	NR	[3]
p.(Gly544Arg) (mosaic)	Missense LoF	M	NR	Early onset epileptic encephalopathy	NR	Severe	NR	NR	NR	NR	[6]
p.(Gly545Asp)	Missense LoF	NR	NR	NR	NR	NR	NR	NR	NR	NR	[6]
p.(Gly545Ser)	Missense LoF	NR	NR	NR	NR	NR	NR	NR	NR	NR	[6]
p.(Gly731Arg)	Missense LoF	M (3)	NR	No	Hypotonia (3/3)	Severe (1/3) profound (2/3)	NR	No	Strabismus (2/3), scoliosis (3/3)	Cortical atrophy (1/2 studied)	[4]
p.(Gly731Val)	Missense LoF	M	11	No	No	No	Mildly delayed	No	Synophrys, straight eyebrows, a high nasal bridge and enophthalmia	Normal	[6]
p.(Gly78Ser)	Missense LoF	M (3)	NR	Yes	NA	Moderate	NR	No	No	NA	[4]
p.(Ile272Val)	Missense LoF	NR	NR	NR	NR	NR	NR	NR	NR	NR	[6]
p.(Ile374Thr)	Missense LoF	NR (2)	NR	NR	NR	NR	NR	NR	NR	NR	[6]
p.(Ile549Leu)	Missense LoF	M	20	Myoclonic, atonic epilepsy which evolved to Lennox–Gastaut syndrome	Wheel-chaired	Severe	Non-verbal	No	NR	Normal	[8]
p.(Ile549Leu)	Missense LoF	NR	NR	NR	NR	NR	NR	NR	NR	NR	[6]
p.(Leu221Val)	Missense LoF	M (4)		Yes (1/4)	Hypotonia (1/4 studied)	Mild (1/4), profound (2/4), not known (1/4)		No	No	Normal (1 studied)	[4]
p.(Leu276Phe)	Missense LoF	M	3	Generalized tonic–clonic seizures	central hypotonia and peripheral spasticity, swallowing difficulties with gastrostomy	Profound	Non-verbal	No	Microcephaly, hyperpigmented lesions on the trunk	Severe cerebral and cerebellar atrophy with thinning of the corpus callosum, mild atrophy of bilateral thalami, abnormal cerebral white matter signal and mild bifrontal cerebral collections	[6]
p.(Leu279Val)	Missense LoF	F	39	Atypical and recurrent tonic–clonic convulsions associated with fevers	Generally increased tone and brisk reflexes	Moderate	Regression	Yes	Broad mouth and short philtrum with minimal micrognathia	NA	[6]
p.(Leu348Val)	Missense LoF	M	5	No	Walking, auditory nerve injury	Moderate	Non-verbal (regression)	Yes	Right groin hernia	Normal	[2]
p.(Lys560Glu)	Missense LoF	NR	NR	NR	NR	NR	NR	NR	NR	NR	[6]
p.(Lys62Arg)	Missense LoF	M	14	Mixed seizure semiology including absences, eye blinking, tonic–clonic seizures, and episodes can be associated with nausea, vomiting and tremor	Balance difficulties	Mild	Delayed	Yes	High palate, macrodontia of the central incisors and restricted extension at the elbows	NA	[6]
p.(Phe319Ser)	Missense LoF	M	20	Drug-resistant epilepsy (tonic–clonic, absence seizures, sometimes provoked by fevers	Hypotonia, walking at 40 months	Moderate	First words at 54 months	Yes	No	Normal	[6]
p.(Phe319Ser)	Missense LoF	M	12	Tonic–clonic seizures often associated with febrile illnesses, absence seizures associated with eyelid myoclonus	Hypotonia, unstable gait	Mild, mainly non-verbal, verbal IQ in the normal range	Speech articulation difficulties	No	No	Normal	[6]
p.(Pro226Leu)	Missense LoF	M	20	Generalized tonic–clonic, absences, sometimes provoked by fevers	Walking at 3.5 years	Moderate	Firs words at 54 months	Yes	No	Normal	[6]
p.(Pro635Arg)	Missense LoF	F	8	Focal and generalized tonic–clonic seizures	NA	Moderate	NA	Yes	Hypertelorism with epicanthal folds and full cheeks	Underdevelopment of the sulci in the left frontal region	[6]
p.(Ser278Arg)	Missense LoF	NR (2)	NR	NR	NR	NR	NR	NR	NR	NR	[6]
p.(Ser283Asn)	Missense LoF	F	37	No	Hypotonia, walking at the age of 2, Progressive spastic diplegia from this age	Moderate	Sentences	Yes	Cushingoid features	NA	[6]
p.(Thr203Ile)	Missense LoF	M	3	No	Hypotonia, walking at 26 months, ataxia	Mild	Some words after the age of 4	Yes	Microcephaly, mild malar flatness, long philtrum, left-sided single palmar crease	Corpus callosum hypoplasia	[6]
p.(Thr203Ile)	Missense LoF	F	11 months	Infantile spasms	Not walking	Yes, not specified the level	Non-verbal	No	No	Delayed myelination	[2]
p.(Val212Gly)	Missense LoF	M (2)	NR	NR	NR	NR	NR	NR	NR	NR	[4,6]
p.(Val275Leu)	Missense LoF	M	4	Absences, infantile spasms, focal seizures	Hypotonia, walking at 4.5 years	Severe	Non-verbal	No	Elongated face, facial hypotonia with an open mouth, full cheeks and micrognathia	Dysgenesis of the corpus callosum	[6]
p.(Val275Met)	Missense LoF	F	NR	NR	NR	NR	NR	NR	NR	NR	[6]
p.(Val275Met)	Missense LoF	M	2	Infantile spasms, tonic– clonic seizures	No ambulation, hypertonia, left-sided unilateral positive ankle clonus and babinski sig	Moderate	Single words	NR	No	Increased T2 signal in white matter, thin corpus callosum	[2]
p.(Val275Met)	Missense LoF	M	6	Myoclonic, tonic, focal seizures	No ambulation	Severe (regression)	Non-verbal	NR	NA	Enlargement of subarachnoid spaces, dilation of bilateral lateral and third ventricle	[2]
p.(Val275Met)	Missense LoF	M	21 months	Tonic, focal, status epilepticus	No ambulation	Severe	Non-verbal	NR	Microcephaly	Dilation of the cerebral ventricles, thin corpus callosum, small hippocampi	[2]
p.(Val275Met)	Missense LoF	M	8	Tonic–clonic, tonic, absence seizures	No ambulation	Profound (regression)	Non-verbal	NR	NA	NA	[2]
p.(Val533Met)	Missense LoF	M	15	Focal	NA	Moderate	NA	Traits	No	NA	[6]
p.(Val536Met)	Missense LoF	M (7), F (2)	NR	Yes (8/8, 1 NR)	Progressive spasticity (2/8, 1 NR)	Mild (4), moderate (2), profound (2) 1 NR	NR	No	Coarse facial features, broad nasal tip, flat midface, prominent ears (4/4 studied)	NA	[4,11]
p.(Val636Met)	Missense LoF	F	4	No	Walking at 23 months	Moderate	Few words	Yes	Microcephaly, down slanting palpebral fissures, telecanthus, small and widely spaced teeth, bilateral clinodactyly of the 5th fingers and slight edema of the dorsum of the feet		[6]
p.(Val92Met)	Missense LoF	F	9	No	No	Yes, not specified the level	Delayed		Synophrys and posteriorly rotated ears	No	[6]
p.(Arg315His)	Missense GoF	F	3	One brief febrile seizure at 34 months	Hypotonia, walking at 21 months	Moderate	Delayed	Yes	No	NA	[6]
p.(Arg315His)	Missense GoF	NR (2)	NR	NR	NR	NR	NR	NR	NR	NR	[6]
p.(Arg364Gly)	Missense GoF	M	27	Absence seizures, focal seizures, and occasionally secondary generalized seizures	normal	Borderline to mild	Normal	No	No	NA	[6]
p.(Arg432Gln)	Missense GoF	NR	NR	NR	NR	NR	NR	NR	NR	NR	[6]
p.(Arg652Thr)	Missense GoF	NR	NR	NR	NR	NR	NR	NR	NR	NR	[6]
p.(Arg718Gln)	Missense GoF	M (1) NR (2)	NR	NR	NR	NR	NR	NR	NR	NR	[6]
p.(Asp29Glu)	Missense GoF	M	10	No	Hypotonia, standing with support, few steps with waking frame	Severe	4 words	Mannerisms/involuntary movements: hand regard, hand, and arm flapping, clapping, smiling, and laughing most of the time	Triangular/trapezoidal face shape, high anterior hairline, prominent forehead, deep-set eyes with straight eyebrows, long/large mouth with thin lips, square, widely spaced teeth	Multiple dilated perivascular spaces, delayed myelination, gray matter heterotopia	[6]
p.(Asp29Glu)	Missense GoF	M	2	Infantile spasms, myoclonic/tonic seizures	Hypotonia, not sitting up unaided, convergent strabismus, nystagmus,	Moderate	Babbling	Mannerisms/involuntary movements: hand regard, hand and arm flapping, clapping, smiling, and laughing most of the time	Triangular/trapezoidal face shape, high anterior hairline, prominent forehead, deep-set eyes with straight eyebrows, long/large mouth with thin lips, square, widely spaced teeth	Normal	[6]
p.(Asp29Glu)	Missense GoF	F	NR	NR	NR	Mild	NR	NR	NR	NR	[6]
p.(Asp34Asn)	Missense GoF (VOUS)	M	9	Focal, atonic, and febrile seizures	Walking at 18 months	Yes, not specified the level	Sentences	Yes	Almond shaped eyes, pointed teeth and a short, upturned nose, rhizomelic limb shortening consistent with Desbuquois Dysplasia	Normal	[6]
p.(Asp621Gly)	Missense GoF	NR	NR	NR	NR	NR	NR	NR	NR	NR	[6]
p.(Asp89Asn)	Missense GoF	M	5	Tonic–clonic seizures	Not sitting independently, no purposeful movements	Profound (regression)	Non-verbal	Yes	Microcephaly and microsomia	complete agenesis of the corpus callosum and cerebellar and brainstem hypoplasia	[8]
p.(Asp89Asn)	Missense GoF	F	13	No	Ataxia	Mild	Delayed	No	Microcephaly, broad nasal root, high palate, fifth finger clinodactyly, pectus carinatum	NA	[6]
p.(Asp89Asn)	Missense GoF	F	3	No	Ataxia, hypertonia, not walking until the age of 2	Mild	Delayed		Microcephaly, right eye internal strabismus and low hairline	Delayed myelination	[1]
p.(Asp89Asn)	Missense GoF	M	Fetus	-	-	-	-	-	Bilateral talipes equino-varus	Agenesis of the corpus callosum, hypoplastic pons, absent septum pellucidum, colpocephaly	[22]
p.(Asp89Asn)	Missense GoF	M	Fetus	-	-	-	-	-	-	Agenesis of the corpus callosum, hypoplastic pons, absent septum pellucidum, colpocephaly	[22]
p.(Ile549Asn)	Missense GoF	F	7	No	Walking at the age of 5	Regression	Regression, single words	Yes	Microcephaly, short palpebral fissures, and a prominent lower lip	Delayed myelination and considerable white matter deficiency with enlarged lateral ventricles and a thin corpus callosum	[6]
p.(Ile646Thr)	Missense GoF	NR (2)	NR	NR	NR	NR	NR	NR	NR	NR	[6]
p.(Ile655Val)	Missense GoF	NR	NR	NR	NR	NR	NR	NR	NR	NR	[6]
p.(Phe238Leu)	Missense GoF	NR	NR	NR	NR	NR	NR	NR	NR	NR	[6]
p.(Phe268Leu)	Missense GoF	F	8	No	Hypotonia, walking at the age of 2, swallowing difficulties	Mild-moderate	Delayed	Yes	Microcephaly	Lipoma, dysgenesis of the corpus callosum	[6]
p.(Pro310Ser)	Missense GoF	F	7	Yes	Hypotonia, walking at the age of 2	Yes, not specified the level	20 words	No	Microcephaly with ridged metopic suture, strabismus, left-sided ptosis, slight ocular telecanthus, lateral fullness of the nose, mild bilateral limited elbow extension, transverse palmer crease	Normal	[6]
p.(Pro369Leu)	Missense GoF	M	13	Yes	Walking at 48 months, wide-based gait, sensorineural hearing loss, optic nerve hypoplasia, swallowing difficulties with gastrostomy feeding	Moderate	Sentences	Yes	Diaphragmatic hernia, left lung hypoplasia, bilateral cryptorchidism, full cheeks, long philtrum, micrognathia, wide nasal bridge, anteverted nares, frontal bossing, exaggerated cupid’s bow, downturned corners of mouth, high anterior hairline, prominent forehead, lagophthalmos, hypertelorism, preauricular pit, posteriorly rotated ears	NA	[6]
p.(Ser105Cys)	Missense GoF	NR (2)	NR	NR	NR	NR	NR	NR	NR	NR	[6]
p.(Ser395Arg)	Missense GoF	F	13	No	Walking at 24 months	Mild	Sentences	No	No	Arachnoid cyst	[6]
p.(Ser69Leu)	Missense GoF (VOUS)	M	5	No	7th cranial nerve palsy, severe myopia, mild ataxia	Severe	Delayed	No	Hypospadias, long eyelashes, synophrys, thick eyebrows, low set ears, short but normal fifth digit phalanges in hands and feet, second toe longer than the hallux on the left side	Normal	[6]
p.(Thr629Ile)	Missense GoF	F	14	No	Walking at the age of 3	Moderate-severe	Delayed, short sentences	No	Long face and arched eyebrows, 2-vessel umbilical cord at prenatal ultrasound	Small remote lacunar infarct and dilated perivascular space, mild prominence of fourth ventricle	[6]
p.(Val317Ile)	Missense GoF	M	5	No	Hypotonia, walking at 5 years	Severe	Non-verbal	Yes	Mildly flat face, everted lower lip, anteverted nares	Partial corpus callosum agenesis (posterior part of the corpus and splenium) with colpocephaly, and mild third ventricle dilation	[6]
p.(Val317Ile)	Missense GoF	M	13	Focal onset frontal lobe hypermotoric epilepsy	Hypotonia, walking at 3.5 years, bilateral optic nerve hypoplasia, strabismus	Moderate	Delayed, first words at the age of 2	No	Elevated finger pads, fifth finger clinodactyly, right preauricular skin tag	Mildly small optic chiasm and optic nerves bilaterally suggestive of optic hypoplasia as well as a dysplastic corpus callosum	[6]
p.(Val317Ile)	Missense GoF	M	18	No	Hypotonia, walking at the age of 3 years, bilateral optic atrophy, swallowing difficulties, gastrostomy feeding	Severe	Delayed, first words at the age of 4	No	Bilateral ptosis, widely spaced teeth, slightly simple ears, malar flatness with long face and pointed chin	Bilateral optic atrophy, hypoplasia of the corpus callosum, prominent subarachnoid spaces	[6]
p.(Val317Ile)	Missense GoF	M	6	No	Hypotonia, feeding difficulties	Severe	First words at 15–18 months, unclear and slow rate speech, limited vocabulary	Traits	NR	Agenesis corpus callosum and hypoplasia of cerebellar vermis	[19]
p.(Val317Ile)	Missense GoF	M	6	Generalized/myoclonic mainly during febrile episodes or intercurrent illnesses	Hypotonia, walking at the age of 20 months, swallowing difficulties for liquids	Moderate-to-severe	Delayed, first word at the age of 2; At the age of 6 years two-words associations	No	No	White matter hyperintensities, temporal horns dysmorphism with hippocampal eversion, dysmorphic corpus callosum and thin optic chiasma.	This study
p.(Val317Phe)	Missense GoF	NR	NR	NR	NR	NR	NR	NR	NR	NR	[6]
p.(Val550Leu)	Missense GoF	F	20	No	Walking at 18 months	Moderate	Mixed receptive-expressive language disorder	Traits	Round face, deep-set eyes, bulbous nose with small nares, partial cutaneous syndactyly of the 2nd and 3rd toes, joint hypermobility	NA	[6]
p.(Asn309Profs)	Truncating	M	52	No	No	Mild-moderate	Delayed	Yes	Prominent ears with simple helix, prominent nose	NA	[6]
p.(Gln663Glyfs)	Truncating	M	18	Generalized seizures	No	Mild-moderate	Delayed	No	Microcephaly, long face, straight eyebrow, thin lips	NA	[6]
p.(Tyr675Ter)	Truncating	M	13	Febrile seizure, tonic–clonic seizures	Hypotonia, walking at 18 months	Severe	Severely delayed, few words	No	No	Bilateral mesial temporal sclerosis, asymmetric, right side more affected	[6]

Legend and abbreviations: ASD: autism spectrum disorder. CAS: Childhood apraxia of speech. F: Female. IQ: Intelligence Quotient. M: Male. MRI: Magnetic Resonance Imaging. In brackets the number of patients reported or studied. NR: Not Reported. NA: Not Available. WAIS-IV: Wechsler Adult Intelligence Scale—Fourth Edition.

**Table 2:** (**A**) summary of the main clinical features of patients with p.(Arg718Trp) mutation. (**B**) summary of the main clinical features of patients with p.(Val317Ile) mutation.

Genetic Variant	Gender	Mutation Origin	Age (Years if not Specified)	Epilepsy	Neurologic Signs	OFC	Strabismus	Intellectual Disability	Language	ASD/Autistic Traits	ADHD	Brain MRI	Reference
(**A**)													
p.(Arg718Trp)	F	De novo	6	Yes, blank stares, cyanosis, and emesis	No	Normal	No	Yes (not specified the level, regression)	Very limited expressive language	Yes, echolalia, stereotyped or repetitive motor movements,	Yes	NA	[6]
p.(Arg718Trp)	F	De novo	9	Hypertonia and staring during hyperpyrexia, erratic clonic movements and leftward head deviation followed by diffuse hypotonia, occurring during febrile and afebrile states	Hypotonia, walking at 24 months, gait instability, pyramidal signs, feeding difficulties	Microcephaly	Yes	Profound	Non-verbal	Yes, midline stereotypies, hand-to-mouth movements, bruxism	No	Bilateral frontal-parietal and periventricular white matter hyperintensities and corpus callosum thinning	This study
p.(Arg718Trp)	F	De novo	8	Absence seizures	Hypotonia			Severe, regression	NR	NR		NR	[11]
p.(Arg718Trp)	F	Unknown	NR	NR	NR			NR	NR	NR		NR	[6]
p.(Arg718Trp)	M	Maternal	1 month	Intractable epilepsy	NR			Severe	NR	NR		NR	[10]
p.(Arg718Trp)	M	De novo	3	Intractable epilepsy	NR			NR	NR	NR		NR	[18]
p.(Arg718Trp)	NR (3)	De novo (1)	NR	NR	NR			NR	NR	NR		NR	[6]
p.(Arg718Trp)	F	De novo	4	Yes	Hypotonia, feeding difficulties			NR	First words at 12–15 months, CAS, dysarthria	Yes		Mega cisterna magna	[19]
(**B**)													
p.(Val317Ile)	M	De novo	5	No	Hypotonia, walking at 5 years	Normal	No	Severe	Non-verbal	Yes	No	Partial corpus callosum agenesis (posterior part of the corpus and splenium) with colpocephaly, and mild third ventricle dilation	[6]
p.(Val317Ile)	M	Inherited from his mother (mosaic carrier)	13	Focal onset frontal lobe hypermotoric epilepsy	Hypotonia, walking at 3.5 years, bilateral optic nerve hypoplasia, strabismus	Normal	Yes	Moderate	Delayed, first words at the age of 2	No	Yes	Mildly small optic chiasm and optic nerves bilaterally suggestive of optic hypoplasia as well as a dysplastic corpus callosum	[6]
p.(Val317Ile)	M	De novo	18	No	Hypotonia, walking at the age of 3 years, bilateral optic atrophy, swallowing difficulties, gastrostomy feeding	Normal	No	Severe	Delayed, first words at the age of 4	No	No	Bilateral optic atrophy, hypoplasia of the corpus callosum, prominent subarachnoid spaces	[6]
p.(Val317Ile)	M	De novo	6	No	Hypotonia, feeding difficulties	Normal	Occasional mild vertical ocular misalignment	Moderate-to-severe	First words at 15–18 months, unclear and slow rate speech, limited vocabulary	Traits	Yes	Agenesis corpus callosum and hypoplasia of cerebellar vermis	[19]
p.(Val317Ile)	M	De novo	6	Generalized/myoclonic mainly during febrile episodes or intercurrent illnesses	Hypotonia, walking at the age of 20 months, swallowing difficulties for liquids	NR	NR	NR	Delayed, first word wat the age of 2; At the age of 6 years two-words associations	No		White matter hyperintensities, temporal horns dysmorphism with hippocampal eversion, dysmorphic corpus callosum and thin optic chiasma.	This study

Legend and abbreviations: ADHD: Attention deficit-hyperactivity disorder. ASD: autism spectrum disorder. F: Female. M: Male. MRI: Magnetic Resonance Imaging. NR: Not Reported. NA: Not Available. OFC: Occipito-frontal circumference.

**Table 3:** List of the main genes associated with phenotypically overlapping syndromes with Rett-like features.

Genes	Mode of Inheritance	Developmental Regression	Purposeful Hand Skills Lost/Absent	Stereotypic Hand Movement	Severe Language Impairment	Gait Abnormalities	ID	Epilepsy	Microcephaly	Breathing Abnormalities	Dysmorphic Features	CNS Abnormalities
*MECP2*	X-linked	+	+	+	+	+	+	+	+	+	−	−
*CDKL5*	X-linked	+/−	+	+	+	+	+	+	+	+	−	−
*FOXG1*	AD	−	+/−	+	+	+	+	+/−	+	−	+	+
*TCF4*	AD	−	+/−	+	+	+	+	+/−	+	+	+	+
*MEF2C*	AD	+	+/−	+	+	+	+	+	+	−	+	+
*STXBP1*	AD, AR	+/−	+/−	+	+	+	+	+	+	−	−	−
*UBE3A*	AD	−	−	+	+	+	+	+/−	+	−	+	+
*PURA*	AD	+/−	+/−	+	+	+	+	+	+	+	+	+
*SLC9A6*	X-linked	+	+/−	+	+	+	+	+	+	−	+	+
*CLCN4*	X-linked	+/−	+/−	+	+	+	+	+/−	+/−	−	+/−	+/−

Legend and abbreviations: AD: autosomic dominant; AR: autosomic recessive; ID: intellectual disability; CNS: central nervous system; +: present; −: absent; +/−: variable.

## Data Availability

The original contributions presented in this study are included in the article. Further inquiries can be directed to the corresponding authors.
